# A Rare Case of Inflammatory Myofibroblastic Tumor Mimicking Fibrous Adhesions Resulting in Bowel Obstruction

**DOI:** 10.1155/2024/7782678

**Published:** 2024-11-06

**Authors:** Stephanie Washburn, Raj Jessica Thomas, Douglas Grider

**Affiliations:** ^1^Edward Via College of Osteopathic Medicine, Blacksburg, Virginia, USA; ^2^Department of Internal Medicine, Cleveland Clinic Akron General, Akron, Ohio, USA; ^3^Department of Internal Medicine, Dermatology Division, Carilion Clinic and Virginia Tech Carilion School of Medicine, Roanoke, Virginia, USA; ^4^Department of Internal Medicine, Edward Via College of Osteopathic Medicine, Blacksburg, Virginia, USA; ^5^Department of Basic Science Education, Virginia Tech Carilion School of Medicine, Roanoke, Virginia, USA; ^6^Dominion Pathology Associates, Roanoke, Virginia, USA

**Keywords:** bowel obstruction, inflammatory myofibroblastic tumor, ischemic colitis, mesenteric mass

## Abstract

Inflammatory myofibroblastic tumor (IMT) is a rare mesenchymal tumors of unknown etiology composed of myofibroblastic cells admixed with inflammatory cells. Presented is a 72-year-old male hospitalized for severe abdominal pain and hematochezia with onset of associated symptoms of fever and sweats a few hours prior to abdominal pain. A computed tomography (CT) demonstrated left colonic thickening interpreted as partial obstruction, gross adhesions, and ischemia. At surgery, marked bowel ischemia from the distal transverse to proximal sigmoid colon was seen with extensive gross adhesions. Histopathology revealed a mesenteric mass chiefly composed of stellate-to-spindled myofibroblastic cells and fibrous adhesions, intermixed with lymphocytes, histiocytes, and plasma cells. The tumor was positive for desmin, smooth muscle actin, and keratin; tumor staging, grade, and postsurgical follow-up were not completed as the patient expired postoperatively. Illustrated is a rare pathologic mimic of ischemic colitis with fibrous adhesions, IMT. Thus, it should not be assumed that fibrous adhesions are always the etiology of obstruction when “adhesions” between sections of bowel are noted radiologically or surgically.

## 1. Introduction

Inflammatory myofibroblastic tumor (IMT) is a rare mesenchymal tumor of unknown etiology composed of myofibroblastic cells admixed with inflammatory cells [1]. Neoplastic in nature, these lesions affect the lung, mesentery, and omentum [1]. Symptomatic presentation may include fever, weight loss, pain, and site-specific complaints [2]. When present within the gastrointestinal tract, symptoms may be consistent with constipation or obstruction [3]. Laboratory abnormalities may include: anemia, thrombocytosis, polyclonal hypergammaglobulinemia, and elevated erythrocyte sedimentation rate (ESR) [2]. Radiographically, the lesion may appear as a targetoid, heterogenous, or homogenous mass characterized by hyper- or hypo-vascularization present with or without calcifications [3]. IMTs exhibit p53, smooth muscle actin, sometimes desmin, and low molecular weight cytokeratin positivity by immunohistochemistry [3, 4]. Alkaline lymphoma kinase (ALK) positivity by immunohistochemistry is noted in 50%–60% of cases when using the D5F3 clone [5, 6].

The differential diagnosis for IMT includes: epithelioid inflammatory myofibroblastic sarcoma (EIMS), liposarcoma, gastrointestinal stromal tumor (GIST), fibromatosis, immunoglobulin G4 (IgG4)-related disease, and inflammatory pseudotumor [3]. These neoplasms rarely metastasize and are considered intermediate-grade neoplasms of soft tissue [3]. Staging of these tumors should be based on primary tumor, regional lymph node involvement, and distant metastasis [3]. Mainstay of treatment is surgical resection with negative margins [3]. Tumors with classical markers can be targeted with antitumoral drugs [3]. Tumors positive for ALK gene rearrangements, reactive oxygen species 1 (ROS1) tyrosine-protein kinase, and neurotrophic tyrosine kinase receptor type 1 or 3 (NTRK1/3) can be treated with antibody-target therapies [3]. IMT has a high recurrence rate following excision and a low occurrence of metastasis [3]. Reported is a case of a 72-year-old male with initial clinical presentation of ischemic colitis and surgical findings consistent with fibrous adhesions in whom further pathologic evaluation revealed an IMT.

## 2. Case Description

### 2.1. Patient Information

Presented is a 72-year-old Caucasian male with a past medical history of cancer of the throat and larynx, type 2 diabetes mellitus, essential hypertension, obstructive sleep apnea, pure hypercholesterolemia, and tobacco abuse. He had no prior abdominal or gastrointestinal complaints and no significant other findings in his medical record.

### 2.2. Clinical Findings

The patient presented to the emergency room with complaints of severe abdominal pain and hematochezia beginning the night prior. He noted onset of associated symptoms of fever and sweats a few hours prior to the abdominal pain. In the emergency department, the patient was noted to be hypotensive and bradycardic, with diffuse abdominal tenderness to palpation on physical examination. Laboratory studies were pertinent for acute liver injury with significant elevation of transaminases. Computed tomography (CT) revealed pancolitis that was suspicious for ischemia versus infectious colitis.

### 2.3. Diagnostic Assessment

**Figure 1:**
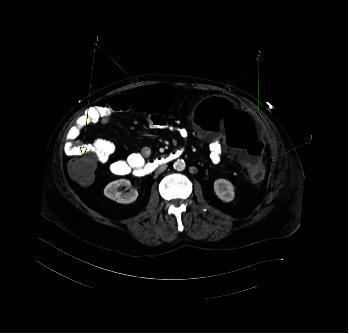
CT scan with normal proximal colon wall thickness (1). Distal transverse colon and proximal descending colon wall thickening and hypoenhancement suspicious for ischemic colitis (2). Point of partial colon obstruction and no visible obstructive colon, omental, or mesenteric mass (3). CT, computed tomography.

The patient was admitted for supportive measures and to monitor for signs of colon perforation. Hospital course was complicated by increased abdominal pain prompting a plain film that demonstrated a possible volvulus. Subsequent CT showed evidence of left colonic thickening possible for obstruction, possible wall dehiscence, and possible ischemia (Figure 1).

### 2.4. Therapeutic Interventions

Diagnostic laparoscopy demonstrated a substantial number of adhesions. Extensive adhesive disease hindered visualization of the abdominal structures, ultimately leading to exploratory laparotomy. Findings revealed full-thickness ischemia of the distal transverse colon to the proximal sigmoid colon with adhesion to the juxtaposed small bowel. Extensive adhesions were noted throughout the abdomen. Sigmoid colon resection with transverse colostomy and small bowel resection was performed. Following surgical intervention, extensive measures were required to maintain hemodynamic stability.

### 2.5. Outcome of Interventions

Ultimately, the patient's family chose to discontinue life-preserving measures, but requested surgical pathology evaluation to understand the cause of death. Pathologic examination in this case involved two distinct specimens—large and small bowel, respectively. The examination of the large bowel demonstrated a tan to tan-red, dusky serosa displaying focal exudate. Several transmural defects were noted with underlying green-brown, dusky, necrotic-appearing mucosa. The small bowel specimen was surfaced by tan to red-brown, dusky, congested serosa involved with a focal exudate and fibrous adhesions. No discrete focal mucosal lesions or abnormalities were noted within the small bowel segment. The pericolonic adipose tissue and mesentery revealed no enlarged lymph nodes.

**Figure 2:**
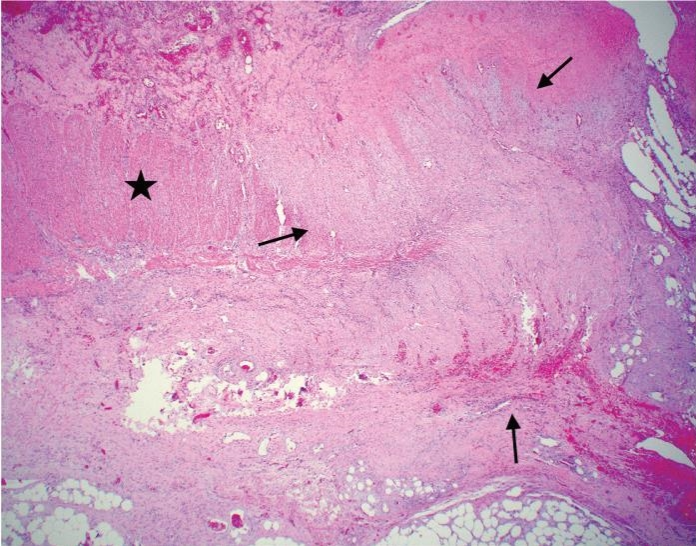
Myofibroblastic cellular proliferation with associated variable fibrosis and intermixed lymphocytes, histocytes, and plasma cells, noted by arrows, infiltrating into the muscularis propria of the colon, noted by the star. Note how the muscularis propria is clearly distinct from the myofibroblastic infiltration (H&E 2x). H&E, hematoxylin and eosin.

**Figure 3:**
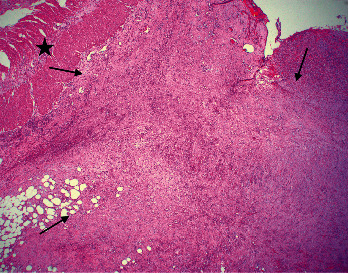
Myofibroblastic cellular proliferation involving the serosa and subserosa of the small bowel denoted by arrows abutting the univolved muscular propria annotated by a star (H&E 2x). H&E, hematoxylin and eosin.

**Figure 4:**
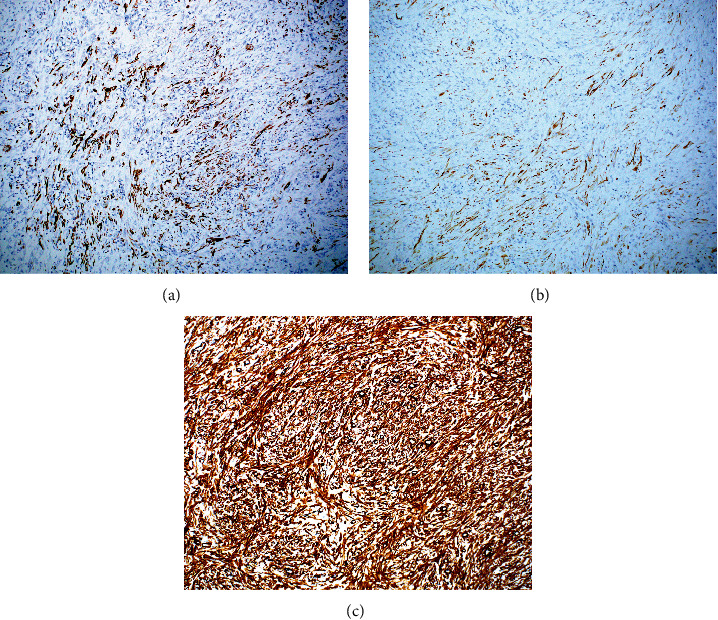
(A) Variable keratin AE1/AE3 positivity in the myofibroblastic proliferation (10x). (B) Variable desmin positivity in the myofibroblastic proliferation (10x). (C) Strong smooth muscle actin positivity in the myofibroblastic proliferation (10x).

Histopathology showed a spindle cell proliferation of myofibroblastic cells with intermixed lymphocytes, histiocytes, and plasma cells within the serosa and subserosa extending into the muscularis propria of the colon with marked fibrosis distinct from the muscularis propria, resulting in partial obstruction secondary to compression and ischemia (Figure 2). The same myofibroblastic cellular proliferation extended into the small bowel subserosa, abutting the muscularis propria (Figure 3). The myofibroblastic nature of the spindled cells was confirmed by immunohistochemistry with positivity for keratin AE1/AE3, desmin and smooth muscle actin, a myofibroblastic cell phenotype (Figure 4A–C, respectively). Immunohistochemical stains for epithelial membrane antigen (EMA) and beta-catenin were negative, ruling out perineurioma and fibromatosis, respectively. S100 was negative, excluding a spindle cell melanoma or peripheral nerve sheath neoplasm. CD117 and CD34 were negative, suggesting against a GIST and solitary fibrous tumor, respectively. The positivity for keratin was noted to be greater in extent than expected for subserosal mesothelial cells, and the corresponding positivity by immunohistochemistry for smooth muscle markers confirmed a myofibroblastic process. Given that the patient expired postoperatively, complete evaluation to include ALK-gene mutation was not pursued because the diagnosis had been confirmed by immunohistochemical analyses. These finding excludes obstruction due to adhesions alone and instead confirms a diagnosis of IMT.

## 3. Discussion

IMTs are rare, with an estimated prevalence of 0.04%−0.7% [7]. The etiology of these tumors remains unknown, though virus-induced trauma, surgery, autoimmune etiology, inflammation, infection, and abnormal response to exogenous stimuli resulting in tumorigenesis have been proposed as possible mechanisms [3]. IMTs are comprised of lymphocytes, plasma cells, histiocytes, fibroblastic, and myofibroblastic cells [8]. In the presented case, microscopic evaluation revealed ischemic changes, transmural necrosis, and fibrous adhesions. Within the serosa and subserosa there was spindled cell proliferation of myofibroblastic cells with variable fibrosis in addition to fibrosis with intermixed lymphocytes, histiocytes, and plasma cells extending into the muscularis propria. The larger spindled myofibroblastic cells were noted to present in a somewhat fascicular pattern and prominent nucleoli were observed in rather large nuclei. On macroscopic exam, IMTs typically present as whorled firm white or yellow infiltrative masses that may include hemorrhage, necrosis, calcification, ulceration, and ossification [1].

The diagnosis of an IMT is one of exclusion, with confirmation of the diagnosis requiring immunohistochemistry. In addition to IMT, the differential diagnosis for unusual fibrosclerosing etiologies of bowel obstruction centered in the mesentery or subserosa includes fibromatosis, sclerosing mesenteritis, IgG4 disease, and GIST.

Myofibroblastic tumors are typically reactive to vimentin, smooth muscle actin, muscle-specific actin, occasionally desmin and low molecular weight keratin [8]. In this case, the tumor cells were positive for keratin AE1/AE3, desmin, and smooth muscle actin. Stains for EMA and beta-catenin were negative, ruling out perineurioma and fibromatosis, respectively. S100 protein and CD117 were also negative, excluding a melanoma. Further, the negative S100 protein immunohistochemical stain excluded a peripheral nerve sheath proliferation, and the negative CD117 suggested against a spindle cell GIST. GISTs are mesenchymal neoplasms with spindle cell or epithelioid histology, typically positive for KIT mutation [9]. CD34 was negative, suggesting this case was not a solitary fibrous tumor, and the typical vasculature pattern of a solitary fibrous tumor was not seen. The histopathology of the tumor cells was not typical for pure smooth muscle differentiation. A fascicular architectural pattern was not noted, nor were the nuclei oblong or “cigar” shaped with perinuclear vacuoles, features known to be seen with smooth muscle cells.

The histological appearance of fibromatosis tends to be comprised of long sweeping fascicles with elongated, slender, spindled cells of uniform appearance and pale cytoplasm set in a collagenous stroma. Minimal cytologic atypia and variable mitotic rate and thin walled and prominent blood vessels with perivascular edema. Cases of fibromatosis usually have nuclear positivity for beta-catenin, though not present in the current case [10].

In the case of sclerosing mesenteritis, microscopic evaluation reveals fibrosis with dense collagen, fat necrosis, perivasculature chronic inflammation, variable focal calcification with minimal atypia, and no or few mitoses. Nuclear beta catenin stain positivity is not present, although IgG4 stains may be positive; fat necrosis is a feature of sclerosing mesenteritis, but is absent in IgG4- related disease [11]. The diagnosis of IgG4-related disease requires at least two of three histologic features: dense lymphoplasmacytic infiltrate, fibrosis usually storiform in character, and obliterative phlebitis [12]. Tissue findings demonstrate >40% IgG4/IgG plasma cell ratio or IgG4+ plasma cells/high power field >30 in surgical specimen or >10 in biopsy specimen [13]. The case presented did not have significant numbers of IgG4+ plasma cells and no obliterative phlebitis.

Despite having low metastatic potential, IMTs are known to have high recurrence rates [3]. Treatment typically involves complete surgical resection with good response to ALK inhibitors in ALK mutated tumors [1]. Complete surgical resection with clear margins is associated with favorable prognosis, while cases of unresectable and/or metastatic disease have limited therapeutic options [14]. In this case, the patient passed away prior to complete evaluation for an *ALK gene* mutation and any further treatment.

## 4. Conclusion

Recognition of this rare mesenchymal tumor is of significance, as clinical manifestation and radiological features may be indistinguishable from a bowel obstruction. IMTs are difficult to diagnose because lower gastrointestinal endoscopy with biopsies is not able to evaluate the subserosa, serosa, or mesentery. Moreover, this case demonstrates an unusual finding of bowel obstruction by a tumorous mass rather than the typical presentation of obstruction by fibrous adhesions. While adhesions are the leading cause of bowel obstruction, they should not always be assumed as the etiology of obstruction when “gross adhesions” between sections of bowel are noted either radiologically or at surgery. The rarity of an IMT should not deter one from making the diagnosis when appropriate, as in this case, and ought to be considered in the differential diagnosis for any mesenteric mass between two portions of the tubular gastrointestinal tract, but especially when there is extension into the wall of the tubular gastrointestinal tract from the serosal side.

## Data Availability

All data, that is case study material, are availabe in the surgical pathology files of Dominion Pathology Associates, Roanoke, Virginia.
